# Preparation of Mechanically Strong Aramid Nanofiber Gel Film with Surprising Entanglements and Orientation Structure Through Aprotic Donor Solvent Exchange

**DOI:** 10.3390/ma18051142

**Published:** 2025-03-04

**Authors:** Zeyu Chen, Chuying Yu, Wenbin Zhong

**Affiliations:** College of Materials Science and Engineering, Hunan University, Changsha 410082, China; zychen@hnu.edu.cn (Z.C.); ychuying@hnu.edu.cn (C.Y.)

**Keywords:** aramid nanofibers, entanglement, aprotic donor, orientation structure

## Abstract

Aramid nanofiber (ANF), a nanoscale building block with a prominently complex structure, can be prepared by splitting poly(p-phenylene terephthalamide) (PPTA) fibers into negatively charged ANFs in a deprotonating manner in a DMSO/KOH solvent system, followed by a subsequent re-protonation process using a proton-donor reagent. There are rare reports regarding the utilization of an aprotic donor reagent to convert deprotonated ANF dispersion into film or gel with a controllable structure and high mechanical properties. In this work, dichloromethane, as an anhydrous aprotic donor solvent, has been introduced into the deprotonated ANF dispersion to replace DMSO, containing PPTA molecules and hydroxyl ions, leading to the gelation of deprotonated ANF dispersions, forming a film (ANF_DCM_) possessing a surprisingly highly entangled and oriented structure, as proven by SEM results. Due to the attenuation of electrostatic repulsion in the dispersion, partially deprotonated ANFs intertwined and cross-linked through π–π conjugation among a large number of benzene rings in PPTA molecules. After treating ANF_DCM_ with water for re-protonation, the as-prepared film (ANF_DCM-W_) exhibited high tensile strength (307.7 MPa) and toughness (74.7 MJ m^−3^).

## 1. Introduction

Since 1960, aramid fiber, a polymer with excellent mechanical properties (strength intensity of 3 GPa), high thermal stability (decomposition temperature exceeding 500 °C), and outstanding chemical resistance, has been widely used in a broad range of fields, such as in the production of fabric, ropes, bulletproof vests, protective layers of optical fiber, and so on [1,2,3,4]. Among numerous aramid fibers with different chemical structures, poly(p-phenylene terephthalamide) (PPTA) fiber has drawn much more attention in research in recent years [4,5,6,7]. Its strong intermolecular hydrogen bonds and intrinsic characteristics endow the fibers with excellent comprehensive performance, but on the other hand, it exhibits extremely poor solubility and is only dissolved in concentrated acid solution, significantly limiting its applications. Considering that breaking the hydrogen bonds between polymer chains through alkaline/organic solvent systems can facilitate their dissolution, PPTA fibers are commonly degraded to deprotonated aramid nanofibers (ANFs) in an alkali/DMSO system, ascribed to the deprivation of the H atom from the N-H group, leading to the breaking of hydrogen bonds among PPTA chains and increasing the electrostatic repulsion between chains [7,8,9,10]. The deprotonated ANF dispersion can then be used to prepare films, coatings, fibers, hydrogels, particles, and composites, which are widely applied in numerous products, such as reinforcement nanocomposites, battery separators, thermal/electrical insulation, and biomimetic tissue [2,11,12,13,14,15,16,17].

The deprotonated ANFs are also commonly re-protonated again to prepare macroscopic materials using protonation reagents including water, ethanol, acetic acid, etc. [5,7,18,19]. For instance, when water is used as the protonation reagent, the prepared ANF film generally exhibits a good tensile strength (100~200 MPa). The use of alcohols (such as methanol or ethanol) as protonation reagents will reduce the mechanical properties of the film, while the use of acetic acid will enhance its mechanical properties. In this sense, the protonation reagents with different proton concentrations affect the aggregation structure and mechanical properties of prepared ANF aerogels [18]. However, it is also found that the tensile strength of ANF films can be regulated by selecting the protonation reagent, but the elongation at break is generally not more than 20%. It is well known that orientation structure and crystallinity are crucial factors in the mechanical properties of ANF films. Back in 1974, for the first time, Northolt and Aartsen [18,19] studied the crystal cell parameters of PPTA by modifying the simulation using an experimental XRD pattern. PPTA fiber exhibits a monoclinic (pseudo-orthorhombic) unit cell (a = 7.87 Å, b = 5.18 Å, c = 12.9 Å (fiber axis), and α = β = 90°, γ ≈ 90°) and possesses *Pn* space-group symmetry. The characteristic 2θ peaks observed around 20.5°, 22.6°, and 29.3° belong to the (110), (200), and (004) diffraction planes, respectively. In previous reports on the XRD patterns of ANF materials, ANF films or aerogels, as protonated by water, generally presented a relatively strong peak around 20.5° and a relatively weak peak around 22.6° [5,19]. Meanwhile, compared with using water as the protonation reagent, when using alcohols (such as methanol or ethanol) in the preparation of ANF aerogels, the peak at around 20.5° was found to be decreased, while the peak at around 22.6° was increased [18]. Compared with PPTA fiber, the relative difference in the intensity of the XRD peak of ANF films or aerogels, protonated by protonation reagents, is generally attributed to the low degree of crystallization, but the possible change in orientation structure and cell parameters of ANF films or aerogels during this process has not been afforded adequate attention in the research. It is necessary to comprehensively evaluate the influence of the degree of crystallization and the orientation structure on the mechanical properties of ANF film.

The microstructure of ANF film or gel significantly affects its mechanical properties [18]. Generally, ANF films or gels are formed by the stacking and cross-linking of nanofibers. A reagent with weak protonation ability (such as methanol, ethanol, etc.) will lead to a smooth microstructure, composed of nanofibers, in the as-prepared ANFs aerogels, while a relatively strong reagent (such as water, acetic acid, etc.) will lead to a granular microstructure. Moreover, a relatively strong type, like acetic acid, will also cause the upper part of the prepared ANF aerogel to present an obviously laminated structure caused by the low cross-linking density between the layers, namely, a weak interaction between them, resulting in the low mechanical performance of the prepared ANF aerogel. Therefore, adjusting the microstructure to increase the cross-linking density in the whole material is considered a feasible way to effectively improve the mechanical performance of ANF films or gels. Additionally, it has recently been reported that the number of entanglements in gels is greatly increased by changing the variety and content of the solvent, cross-linker, and initiator, resulting in the enhancement of the toughness and stiffness of the gels [20]. Moreover, the weak protonation reagents (such as methanol or ethanol) commonly used for preparing ANF films are mutually soluble with water, which may introduce trace water during the gelation process, hence affecting the microstructure and orientation structure during the preparation process. From this view, an aprotic donor solvent that cannot provide protons and is immiscible with water will benefit the controllable formation of different microstructures, such as entanglements and orientation structures, in the as-prepared ANF films, without the interference of water action. In other words, the creation of ANF films with excellent mechanical properties will be predicted by treating deprotonated ANF dispersions with aprotic donor solvent. In this work, an anhydrous aprotic donor, dichloromethane, was introduced into an ANF dispersion to replace DMSO, which was gelled and then converted into a film with high tensile strength and toughness.

## 2. Experimental Setup

### 2.1. Materials

Poly(p-phenylene terephthalamide) (PPTA) fibers (Kevlar 1000D) were obtained from Dongguan Sovetl Co., Ltd., Dongguan, China. Dimethyl sulfoxide (DMSO), dichloromethane (DCM), and dichloroethane (DCE) were all AR grade and purchased from Sinopharm Chem. Reagent Co., Ltd., Shanghai, China, and potassium hydroxide (KOH) was purchased from the same source. All chemical reagents were used without any further purification.

### 2.2. Preparation of Aramid Nanofiber (ANF) Dispersion

The deprotonated aramid fiber (ANF) dispersion (1.0 wt.%) was prepared according to the literature: 1.5 g of KOH and 1 g of PPTA fiber were successively added to 100 mL DMSO under stirring [7]. The mixture continued to be stirred at 30 °C for 7 days to prepare a dark-red ANF dispersion.

### 2.3. Preparation of ANF_DCM_, ANF_DCM-W_, ANF_W_, and ANF_DCE-W_ Film

The ANF_DCM-W_ film was prepared using the following procedure: 1.5 mL ANF dispersion (1.0 wt%) was uniformly cast on a glass slide (25.4 mm × 76.2 mm), which was then immersed in dichloromethane in a Petri dish to initiate the gelation process. The Petri dish was covered and placed in suitable conditions. In the following two days, dichloromethane was replaced carefully with fresh solvent twice every 24 h. After that, dichloromethane in the Petri dish was removed, and the gel sample was dried naturally at room temperature to obtain the ANF_DCM_ film. Subsequently, the collected ANF_DCM_ film was re-immersed in deionized water for two days, and the water was replaced every 8 h. After that, the film sample was dried naturally at room temperature and labeled as ANF_DCM-W_ film. Following the same procedure, the ANF_W_ film was prepared with solvent replaced with water, and the ANF_DCE-W_ film was prepared with solvent replaced with dichloroethane (DCE) and water.

### 2.4. Material Characterization

Scanning electron microscopy (SEM) images of the sample were obtained using a Hitachi S-4800 (Hitachi, Tokyo, Japan). X-ray photoelectron spectroscopy (XPS) measurements were carried out on a Thermo ESCALAB 250Xi (Thermo Fisher Scientific, Waltham, MA, USA). X-ray diffraction (XRD) data were collected with the D8-ADVANCE. The ^13^C solid-state nuclear magnetic resonance spectra were recorded on a Bruker AVANCE III HD 400 spectrometer (Bruker, Billerica, MA, USA), operating at 400 MHz, employing a double-tuned solid-state probe equipped with 4 mm (o.d.) spinners. The ^13^C CP/MAS results were recorded using a spin rate of 12 kHz. Mechanical properties of the as-prepared films were obtained using an AGS-X 500 N (Shimadzu, Tokyo, Japan) mechanical testing system at a tensile rate of 1 mm min^−1^, wherein 3 paralleled samples were tested for each set, and the results were averaged for records. Atomic force microscopy (AFM, Oxford MFP-3D origin, Oxford instruments, Oxford, UK) was used to characterize the morphology of the samples. The molecular weight distribution of deprotonating ANF dispersion was characterized by gel permeation chromatography (GPC, DMSO as mobile phase, PL-GPC50, Agilent, Santa Clara, CA, USA). UV-vis absorption spectra were recorded on a UV-2250 UV-vis spectrophotometer (Shimadzu, Tokyo, Japan). The thermal properties of the as-prepared samples were investigated using thermogravimetric analysis (TGA) and differential scanning calorimetry (DSC) with a Hitachi STA300 (Hitachi, Tokyo, Japan). Fourier transform infrared (FTIR) spectra of the samples were characterized on a Thermo iS50 spectrometer (Thermo Fisher Scientific, Waltham, MA, USA). The contact angles were characterized using an optical contact angle meter (OCA 15EC, Dataphysics Instruments, Filderstadt, Germany).

## 3. Results and Discussion

**Scheme 1:**
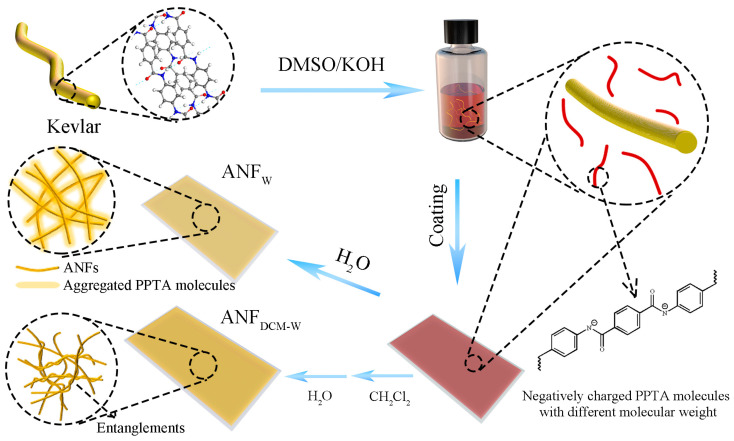
Illustration of fabrication process of ANF_DCM-W_ film with dichloromethane as solvent to exchange DMSO and ANF_W_ film by using water to exchange DMSO.

The fabrication of the ANF_DCM-W_ and ANF_DCE-W_ films is illustrated in Scheme 1. Briefly, it involves three procedures: (1) Deprotonation of Kevlar fibers to deprotonated ANF dispersion in the DMSO/KOH system. It is sort of a degradation/de-assembly process: the original Kevlar fibers partially break down and release negatively charged PPTA macromolecules with different molecular weights. (2) Gelation by re-protonation. The prepared ANF nanofluid dispersion is re-protonated by protonation reagents such as aprotic donor dichloromethane or strong proton donor water. In this process, ANF dispersion is initiated to gel, namely, ANF_DCM_ and ANF_W_ gels in the present work. (3) Filmation after further protonation. In this process, the collected ANF gels were naturally dried and then further treated (re-protonated) with water. In the final collected ANF_DCM_ and ANF_W_, different protonation reagents used in the former step cause different microstructures, namely, aggregated PPTA macromolecules in the ANF_W_ gel or more entanglements in the ANF_DCM_ gel.

### 3.1. Tensile Strength and Fracture Toughness of Films

**Figure 1:**
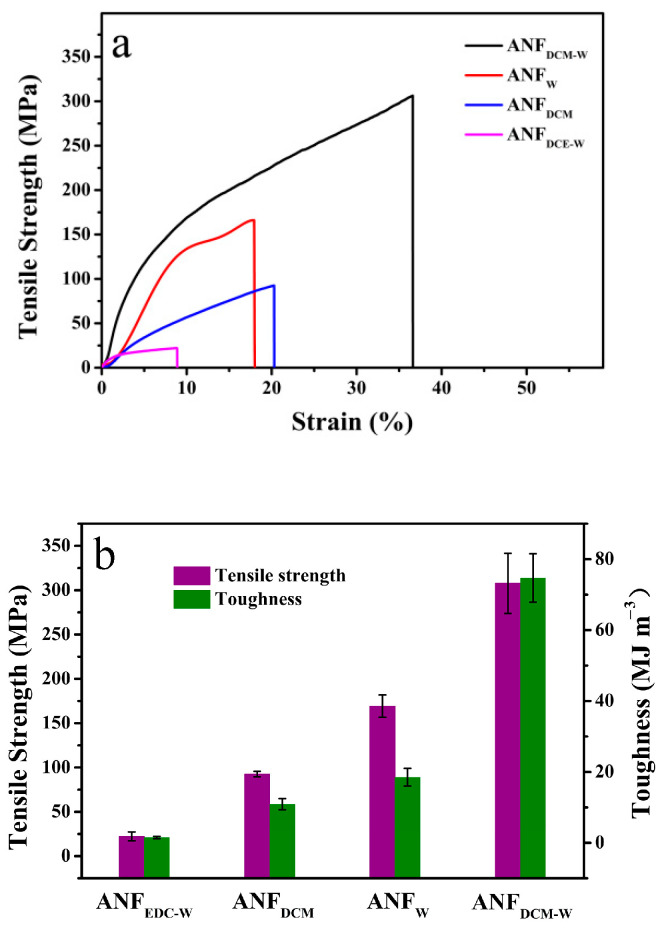
(**a**) Typical tensile stress–strain curves and (**b**) comparison of the tensile strength and toughness of ANF_DCM-W_, ANF_W_, ANF_DCM_, and ANF_DCE-W_ film.

The mechanical properties (including tensile strength and fracture toughness) of the as-prepared films were measured (results shown in Figure 1 and Figure S1). The ANF_DCM-W_ film exhibits about twice the tensile strength (307.7 ± 34.0 MPa) of the ANF_W_ film (169.2 ± 12.6 MPa), about twice the elongation at break (36.6%) of the ANF_W_ film (18.1%), and about four times the toughness (74.7 ± 6.8 MJ m^−3^) of the ANF_W_ film (18.5 ± 2.5 MJ m^−3^). Meanwhile, the tensile strength and toughness of the ANF_DCM_ film (92.5 ± 3.1 MPa and 10.9 ± 1.6 MJ m^−3^) and the ANF_DCE-W_ film (22.2 ± 5 MPa and 1.5 ± 0.3 MJ m^−3^) are fairly low. The obtained ANF_DCM-W_ film exhibits excellent tensile strength and toughness, surpassing those of all reported pure ANF films (see Table S1 for details) and approaching those of ANF-based composite films, such as mica-based nanopaper with a 3D aramid nanofiber framework, which presents a high tensile strength of 175 MPa and ultra-high toughness of 109 MJ m^−3^ [6].

In order to reveal the crucial factors to the different mechanical properties of these ANF-based films, their chemical structure, orientation structure, and microstructure were characterized and analyzed.

To reveal the factors crucial to the different mechanical properties of these ANF-based films, their chemical structure, orientation structure and microstructure were characterized and analyzed.

### 3.2. Chemical Composition of Films

**Figure 2:**
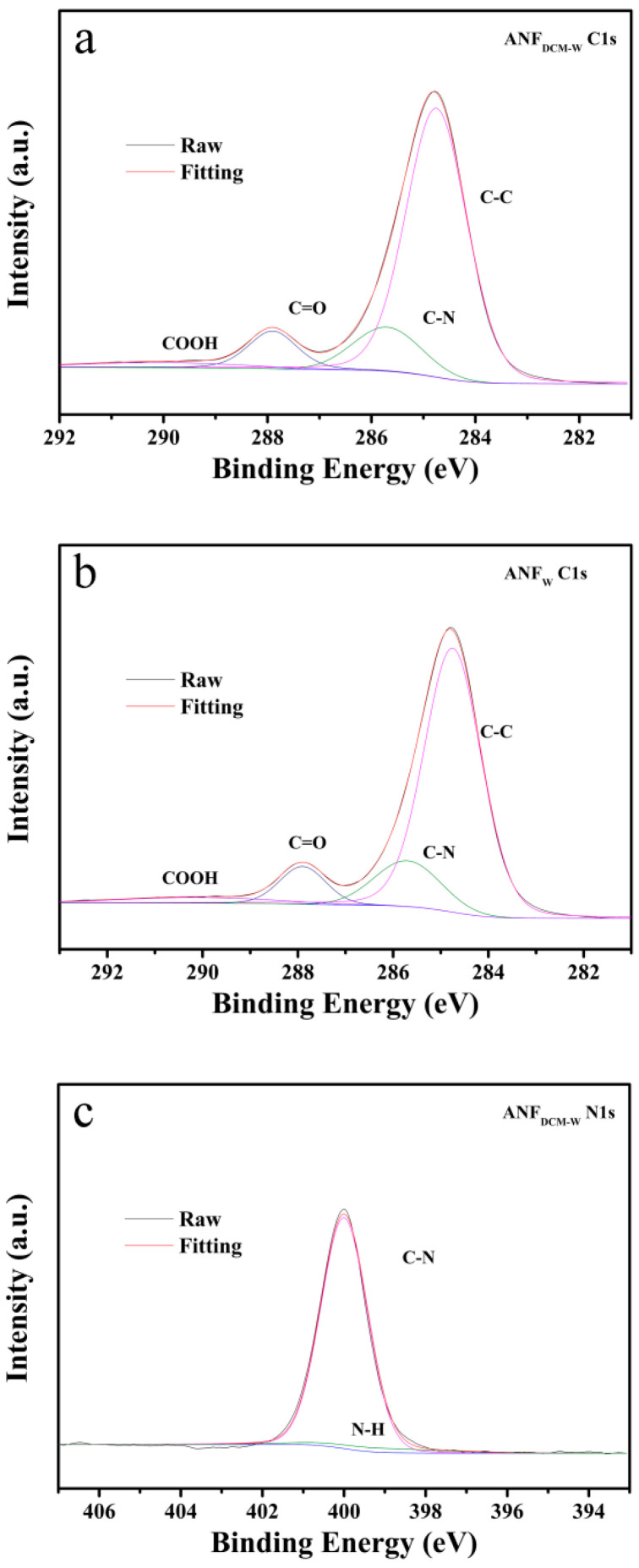

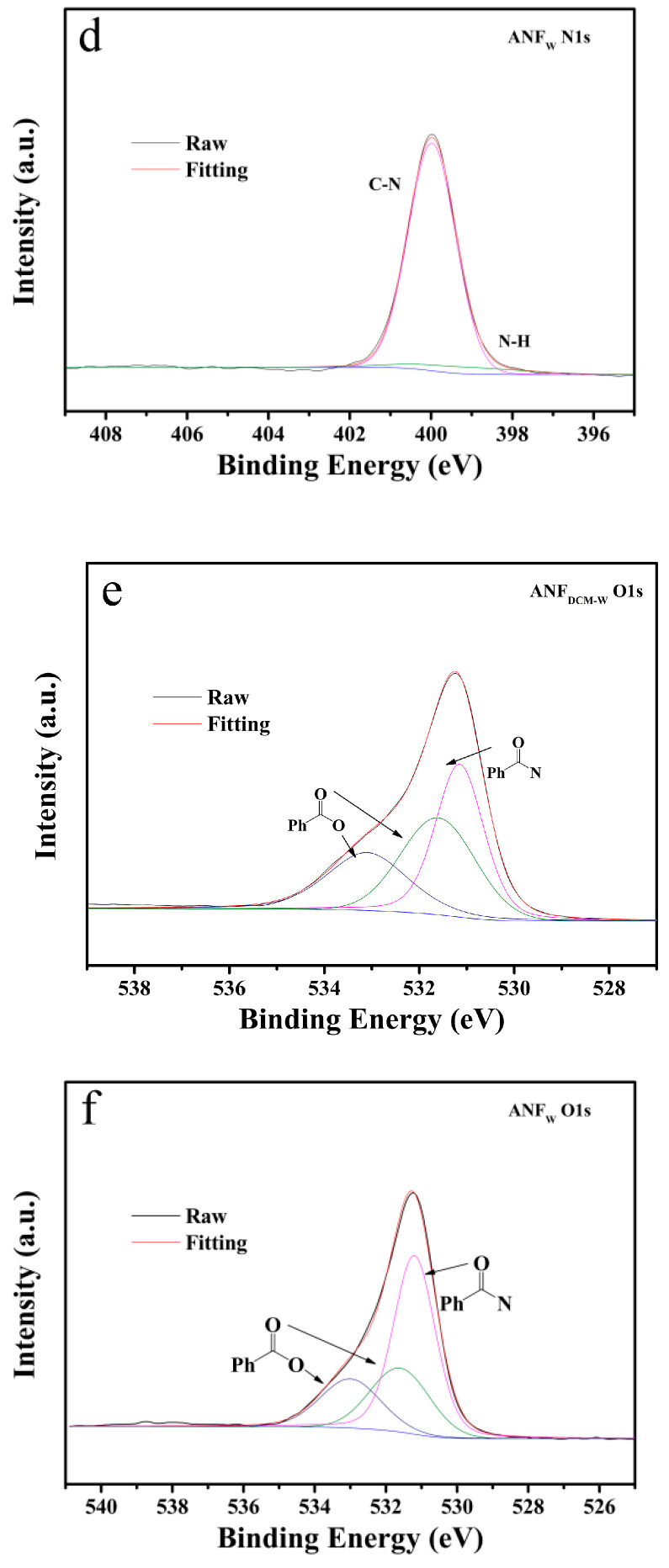
Decomposed C1s spectra of (**a**) ANF_DCM-W_ and (**b**) ANF_W_. Decomposed N1s spectra of (**c**) ANF_DCM-W_ and (**d**) ANF_W_. Decomposed O1s spectra of (**e**) ANF_DCM-W_ and (**f**) ANF_W_.

As alkyl bromides and aralkyl chlorides may react with deprotonated PPTA molecules to obtain *N*-substituted PPTAs [21], in the current case of DCM and DCE, the chemical structures of the ANF_DCM-W_ film and the ANF_W_ film were firstly characterized using ^13^C solid-state nuclear magnetic resonance (^13^C SSNMR) (Supplemental Figures S2 and S3). The results demonstrate that both the ANF_DCM-W_ and ANF_W_ films show a similar single peak at 165.9 ppm and multi peaks at 115~143 ppm, attributed to carbonyl and benzene, respectively. The multi peaks at −1~23 ppm and 236~260 ppm represent the spinning sidebands of benzene carbon. The X-ray photoelectron spectroscopy (XPS) results of the ANF_DCM-W_ and ANF_W_ films (Figure 2) confirm their similar chemical structure, and their C1s, O1s, and N1s spectra demonstrate the same status, too. The element contents analysis of these films (Supplemental Figure S4 and Tables S2–S5) further proves this. Finally, the FTIR spectra of the ANF_DCM-W_ and ANF_W_ films also confirm that they have the same chemical structure (Supplemental Figure S5). Therefore, this may suggest that the different exchange solvents, either dichloromethane or water, used in the present work did not affect the chemical structure of the final obtained films.

### 3.3. Orientation Structure in the Films

**Figure 3:**
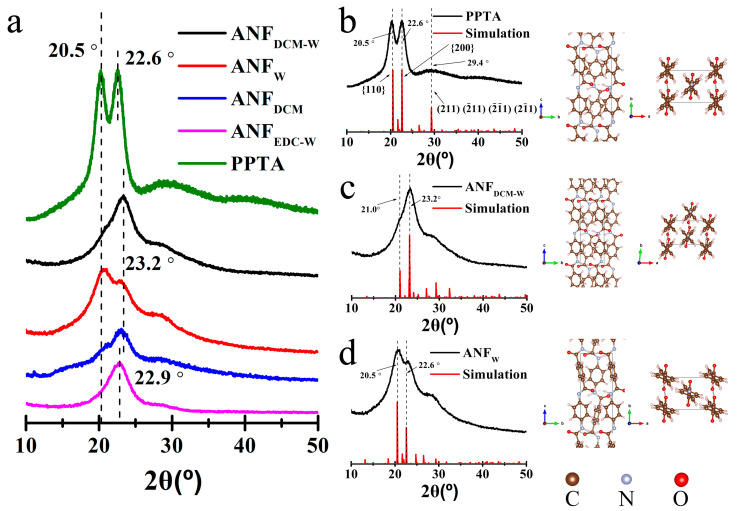
(**a**) XRD spectra of PPTA, ANF_DCM-W_, ANF_W_, ANF_DCM_, and ANF_DCE-W_, (**b**) simulated orientation structure of PPTA, (**c**) simulated orientation structure of ANF_DCM-W_, and (**d**) simulated orientation structure of ANF_W_.

XRD patterns of PPTA fiber, ANF_DCM-W_ film, and ANF_W_ film were then determined to analyze the changes in possible orientation structure (Figure 3). As reported previously [20,22], PPTA fiber exhibits strong characteristic diffraction 2θ peaks at around 20.5° and 22.6°, and weak peaks at 29.3° and 41.2°. The ANF_W_ film exhibits a strong diffraction peak at 20.5° and weak peaks at around 22.6° and 28.2°. However, the ANF_DCM-W_ film exhibits a strong diffraction peak at 23.2° and weak peaks at around 20.5° and 28.2°. According to the cell parameter and atomic position provided in the literature [22,23], simulation of XRD was carried out by using VESTA software (Ver. 3.5.7) to verify whether the molecular model corresponds to PPTA fibers in our work (see Supplemental Tables S6–S8 for details). The simulated XRD pattern exhibits two strong peaks at 20.5° and 22.6° corresponding to the diffraction of {110} and {200} planes, and a strong peak at 29.4° relating to the diffraction of (211), ($\bar{2}$11), ($\bar{2}\bar{1}$1), and (2$\bar{1}$1) planes, which corresponds to the experimental XRD pattern of PPTA. These broadening peaks are perhaps due to the small crystalline grain size and the slight change in cell parameters or orientation structure (Figure 3). By adjusting the molecular model, it can be found that changing the rotation angle of benzene will influence the relative diffraction intensity of {200} planes and the diffraction peak position of (211), ($\bar{2}$11), ($\bar{2}\bar{1}$1), and (2$\bar{1}$1) planes. When the angle between the amide plane and the phenylene plane of the *p*-phenylenediamine segment is 26°, and the angle between the amide plane and the terephthalic segment is 9°, the simulated XRD pattern is consistent with the XRD pattern of the ANF_W_ film. It is implied that the formation of the ANF gel film from ANF dispersion depends on the introduction of protons to interact with –N^−^– to form –NH–, subsequently developing hydrogen bonds with the carbonyl group. The interplanar angles of benzamide and acetanilide can rotate freely, which reduces the π–π conjugation between PPTA molecular chains and the degree of order. When dichloromethane is selected as the solvent exchange reagent, ANF dispersion can also self-assemble into a gel. Because of the residual KOH in the gel, the XRD pattern of the ANF_DCM_ film derived from the gel exhibits a strong diffraction peak at 23.2° and three weak peaks at about 15.2°, 20.5°, and 28.2°. After the ANF_DCM_ film was treated with water, the peak at 15.2° in the XRD pattern of ANF_DCM-W_ disappeared, suggesting the removal of KOH. Regarding the addition of dichloromethane to replace the KOH/DMSO, due to the weak proton-donating capability of dichloromethane, PPTA molecular chains are mainly just re-arranged by π–π conjugation action. In the subsequent step using water to immerse the ANF gel, hydrogen bonds among PPTA macromolecules are formed, and residual KOH is washed out. By adjusting the cell parameters and orientation structure, it was found that all diffraction peaks of the ANF_DCM-W_ film could be satisfactorily indexed by assuming a monoclinic unit cell with dimensions a = 7.7 Å, b = 4.7 Å, c = 12.9 Å (fiber axis) and α = β = 90°, γ = 84°, the angle between the amide plane and the phenylene plane of the *p*-phenylenediamine segment is 12°, and the angle between the amide plane and the terephthalic segment is 16°. The distance of N…O and the angle of N-H…O in the simulated orientation structure of ANF_DCM-W_ are 2.62 Å and 155.1°, which are less than those of ANF_W_ (3.04 Å and 155.5°), and the distance between the two benzene rings of two adjacent chains in ANF_DCM-W_ is also shorter than that of ANF_W_, indicating that the interaction strength of ANF_DCM-W_ is higher than that of ANF_W_. The XRD pattern of the ANF_DCE-W_ film is similar to that of ANF_DCM-W_, except the diffraction peak of {200} planes shifts to 22.9°.

### 3.4. Morphology and Microstructure

**Figure 4:**
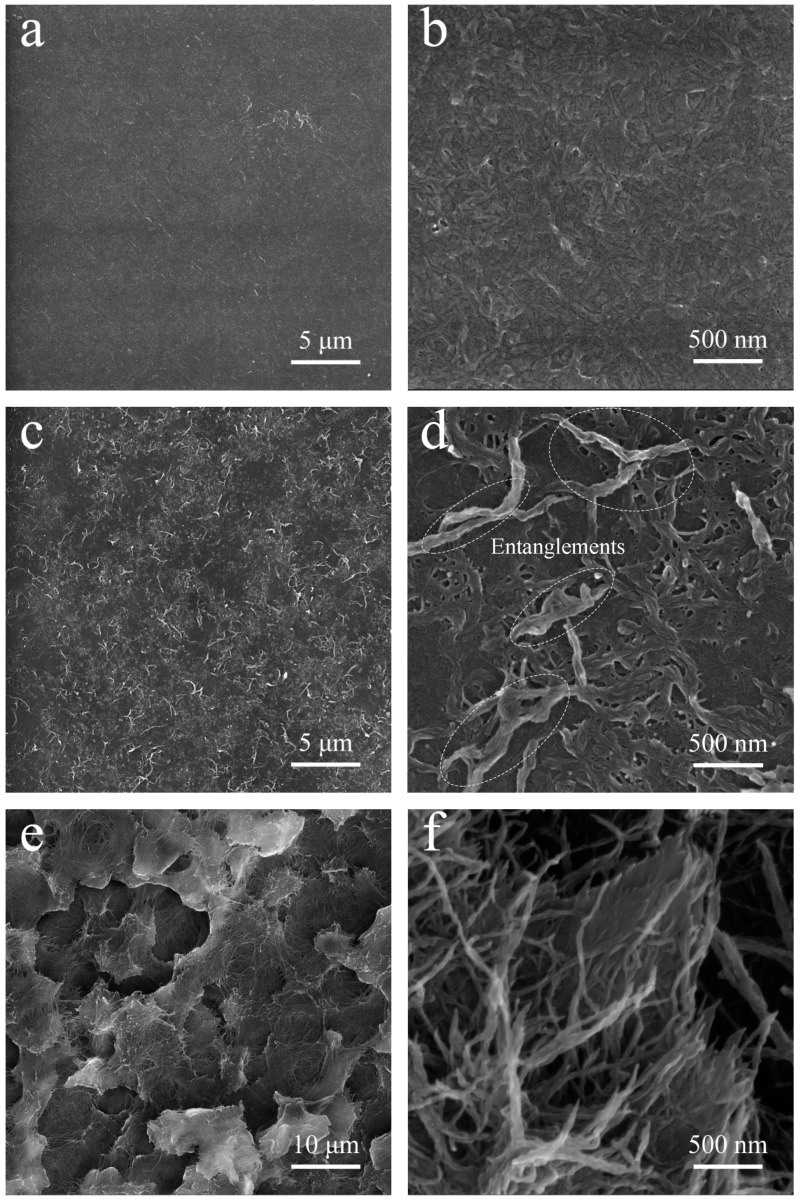
The internal microstructure of (**a**,**b**) ANF_W_, (**c**,**d**) ANF_DCM-W_, and (**e**,**f**) ANF_DCE-W_ film.

Based on the above results, it can be found that the chemical structure and orientation structure of the ANF_DCM-W_ and ANF_DCE-W_ films are similar, but the tensile strength of the ANF_DCM-W_ film is one order of magnitude higher than that of the ANF_DCE-W_ film. To further determine the reason for the different mechanical properties of the ANF_W_, ANF_DCM-W_, and ANF_DCE-W_ films, the surface morphology and microstructure of the films were characterized using SEM (Supplemental Figure S6). The ANF_W_ film exhibits a regular and granular surface with many nanofibers with a small diameter (about 10–20 nm, Supplemental Figures S6a,b and S7a). However, the surface of the ANF_DCM-W_ film is smooth and contains abundant nanofibers (the diameter is about 20–40 nm, Supplemental Figures S6c,d and S7b), and the nanofibers were entangled with each other, forming coarser fibers. Regarding the ANF_DCE-W_ film, its surface is uneven and bulged, and the diameter of the nanofibers is about 15–30 nm (Supplemental Figures S6e,f and S7c), which is lower than the diameter of the nanofibers in the ANF_DCM-W_ film. These films were peeled off by tape to observe the internal morphology (Figure 4). The interior of the ANF_W_ film also exhibits a highly smooth structure as its surface, indicating a laminar structure and weak interaction between layers (Figure 4a,b), but the interior of the ANF_DCM-W_ film is quite rough, with abundant protruding and twisted fibers, whose diameter is about 50~160 nm and composed of smaller fibers (20–30 nm in diameter) (Figure 4c,d, and the illustrated structure shown in Scheme 1). The sliding of twisted nanofibers may cause this disengagement of large fibers. Meanwhile, a fractured surface formed by the breaking of nanofibers can be observed, which indicates the existence of a high degree of internal cross-linking and strong interaction in the ANF_DCM-W_ film. Regarding the ANF_DCE-W_ film (Figure 4e,f), a much more irregular stacking structure can be observed. The entangled nanofibers randomly aggregate into irregular flakes, which then stack irregularly to form a looser structure than observed with ANF_w_ or ANF_DCM-W_, which may directly impact the mechanical properties of the final ANF_DCE-W_ film.

**Figure 5:**
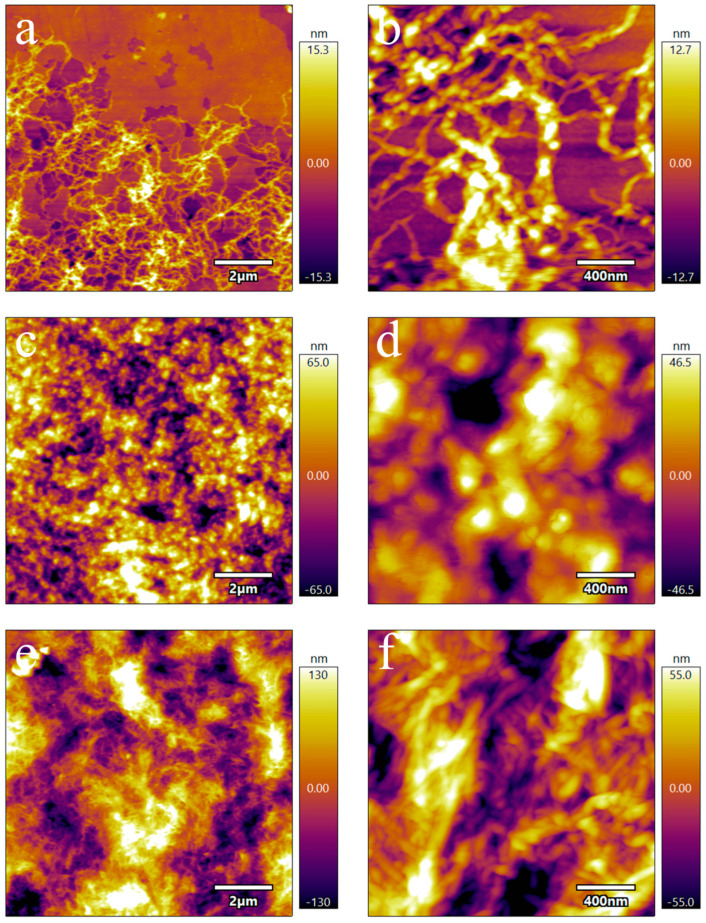
AFM images of (**a**,**b**) ANFs dispersion, (**c**,**d**) ANF_W_ film and (**e**,**f**) ANF_DCM-W_ film.

During the SEM sample preparation, gold was sputtered on the sample’s surface to improve the surface conductivity. To eliminate the gold influence on the surface morphology, the more authentic microstructure of the ANF_DCM-W_ and ANF_W_ films was measured with atomic force microscopy (AFM) (Figure 5). The morphology of ANFs in ANF dispersion was observed, and the diameter of the ANFs is about 10–20 nm (Figure 5a,b), which is consistent with the literature [2,5,7]. A particle-like accumulation structure can be observed in the AFM images of the ANF_W_ film (Figure 5c,d), which may be attributed to the self-cross-linking of ANFs to form a curly structure when water is used as the protonation reagent. However, the ANF_DCM-W_ film shows an entangled and cross-linked structure of nanofibers (Figure 5e,f). The abundant entanglements of nanofibers provide the as-prepared ANF_DCM-W_ film with excellent tensile strength and toughness.

**Figure 6:**
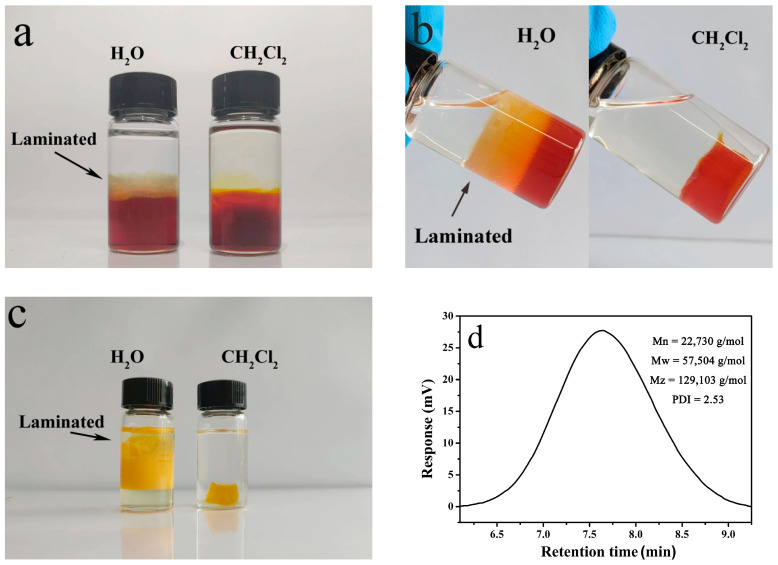
The images of gelation process of ANF_W_ gel and ANF_DCM-W_ gel: (**a**) 2 h, (**b**) 24 h, (**c**) 72 h, (**d**) GPC characterization of ANFs dispersion.

In the present research, the gelation process of ANF dispersion in water and dichloromethane was traced and compared at time intervals of 2 h, 24 h and 72 h (Figure 6) to better understand the reasons behind the different structures of the ANF_DCM-W_ and ANF_W_ films. After water was added for 2 h, numerous yellow, fluffy, thin layers were formed that floated up and congregated in the upper part of the dispersion. When dichloromethane was added, a dark-red gel was formed in the upper part of the dispersion (Figure 6a). Another group of samples was tilted at a certain angle to observe the phenomenon of the gel formed protecting the dispersion (Figure S8). When the tilt angle reached about 90° and 120°, the gel formed by water slipped to the bottleneck, while the gel formed by dichloromethane could maintain its original state. The results suggest that the gel prepared with dichloromethane possesses high mechanical strength and density. When the gelation time lasts for 24 h, it can be observed that the ANF_DCM_ gel shrinks and sinks to the bottom of the bottle, and the ANF_W_ gel is formed from the top to the bottom of the bottle (Figure 6b). In addition, the formed gel is still red, indicating that the gelation process has not finished. After gelation for 3 days, the ANF_W_ gel turns into a yellow and red dispersion, indicating that the gelation process is complete. Simultaneously, the ANF_DCM_ gel also turns yellow, which could be attributed to the solvent effect of dichloromethane (Figure 6c). Additionally, the as-formed ANF_W_ gel floats in the bottle, indicating that its density is close to that of the solution. Meanwhile, the ANF_DCM_ gel further shrinks at the bottom of the bottle, indicating that the ANF_DCM_ gel has a dense structure, and its density is higher than that of the dichloromethane/DMSO mixture.

### 3.5. Formation Mechanism of Gel Films

To further understand the difference in the formation mechanism of gels prepared with dichloromethane and water, we created a GPC spectrogram of ANF dispersion using DMSO as the mobile phase, as shown in Figure 6d, wherein molecules with different molecular weights can be seen. These molecules should be PPTAs with negatively charged aromatic amines or aromatic carboxylic acid anions along their backbones, which are formed by the hydrolysis of PPTA molecules during the chemical exfoliation process. Due to the electrostatic repulsion, partially deprotonated ANFs with negative charges disperse in the DMSO; the abundant negatively charged PPTA molecules along the ANF fibers and the hydroxyl ions formed by the solvent effect of KOH/DMSO cause them to be dissolved in the dispersion. Since the negatively charged PPTA molecules with different molecular weights can be dissolved in dichloromethane but are insoluble in water, with the addition of dichloromethane, negatively charged PPTA molecules with different molecular weights in ANF dispersion can be transferred to the dichloromethane solvent. To verify the solubility of PPTA molecules in dichloromethane or water, the UV-vis absorption measurements were performed with the supernatants extracted from the ANF_DCM_ and ANF_W_ gel systems, respectively, and the absorption spectra are shown in Supplemental Figure S9. An obvious absorption around 410 nm can be clearly seen with the supernatant extracted from the ANF_DCM_ gel system but not with the supernatant extracted from the ANF_W_ gel system, which indicates the existence of PPTA molecules in dichloromethane but not in water. When water is used as the solvent exchange reagent, both the partially deprotonated ANFs and different molecular weight PPTA molecules are rapidly and completely protonated, and then cross-linked or self-cross-linked into the gel. However, the layer-by-layer growth of the ANF_w_ gel causes it to float in the water/DMSO mixture (Figure 6a–c), resulting in a low cross-linking density among layers, which greatly reduces the mechanical properties of the final ANF_w_ film that dried from the gel. When the dichloromethane is used as the solvent exchange reagent, most deprotonated PPTA molecules and hydroxyl ions in the dispersion transfer to dichloromethane (Figure S9), and in addition to this, the electrostatic repulsion is diluted in the gel, while the remaining partially deprotonated ANFs will be curled because of their non-uniform charge distribution and the increase in entropy. Accompanied by the cross-linking through π–π conjugation, the deprotonated ANFs become entangled and twist into the large-size nanofibers (Figure 4c,d and Figure 5e,f), greatly increasing the contact area among the fibers, which could help improve the tensile strength and toughness of the formed film. When the protonation reagent is introduced subsequently, the orientation structures in the ANF_DCM-W_ films are unexpected because of the good compatibility between dichloromethane and negatively charged PPTA molecules (Figure 3), resulting in a shorter π–π conjugation distance and hydrogen bond distance, thus improving its mechanical properties. Regarding the ANF_DCE-W_ gel film, its gelation process is similar to that of the ANF_DCM-W_ gel film, but the interior irregular stacking produces a loose structure (Figure 4e,f and Figure S4e,f), which greatly reduces the cross-linking effect between layers, resulting in fairly low tensile strength and toughness.

### 3.6. Thermal Properties and Wettability

The thermal properties of the ANF_DCM-W_ and ANF_W_ films were evaluated with differential scanning calorimetry (DSC) and thermogravimetric analysis (TGA). The typical DSC curves of the ANF_DCM-W_ and ANF_W_ films are shown in Supplemental Figure S10a. All the films have no obvious melting or glass transition temperature, which is consistent with the literature [24]. The thermal decomposition temperature of the film is determined with TGA, and its maximum decomposition temperature is derived from the corresponding differential thermal gravity (DTG) curve, as shown in Supplemental Figure S10b,c. The first region (around 100 °C) represents the evaporation of water molecules trapped in the film, and it can be observed that the mass loss of the ANF_DCM-W_ film in this region is higher than that of the ANF_W_ film. The second region corresponds to hydrogen bond breaking and degradation of the polymer. The maximum decomposition temperatures of the ANF_DCM-W_ and ANF_W_ films are 496 °C and 513 °C. The water contact angle of the ANF_DCM-W_ and ANF_W_ films is used to evaluate their wettability (Supplemental Figure S11). The ANF_DCM-W_ film presented a 42.1° water contact angle, which was lower than that of the ANF_W_ film (57.8°), and the surface energy of the ANF_DCM-W_ and ANF_W_ films is 55.31 and 42.88 mN/m, respectively. The water adsorption of the ANF_DCM-W_ and ANF_W_ films was tested, and the results of the ANF_DCM-W_ and ANF_W_ films were 13.3 and 11.3.

## 4. Conclusions

In conclusion, the ANF_DCM-W_ film was prepared by deprotonating ANF dispersion with dichloromethane as a solvent exchange reagent and washed with water for re-protonation and removal of impurities. In the absence of protons, the different molecular weight PPTA molecules and hydroxyl ions in the KOH/DMSO system can be removed with dichloromethane, which decreases the electrostatic repulsion and cross-linking between ANFs and PPTA molecules in the system. The partially deprotonated ANFs can curl because of their non-uniform charge distribution and the increase in entropy and intertwine and interact through π–π conjugation among benzene rings in PPTA molecules. Compared with the ANF_W_ film, the ANF_DCM-W_ film exhibits a different orientation structure with a strong 2θ peak at 23.2° and a dense structure constructed with abundant entanglements and interpenetration of nanofibers. Therefore, the as-prepared ANF_DCM-W_ film possesses excellent tensile strength (307.7 MPa) and toughness (74.7 MJ m^−3^). This work provides a new strategy for the preparation of high-performance ANF-based materials using ANF dispersion, and the as-prepared ANF_DCM-W_ film exhibits potential prospects in battery separation, thermal insulation, and so on.

## Data Availability

The original contributions presented in this study are included in the article/Supplementary Materials. Further inquiries can be directed to the corresponding author.

## Supplementary Materials

The following supporting information can be downloaded at: https://www.mdpi.com/article/10.3390/ma18051142/s1, Figure S1: Tensile stress–strain curves of all films; Figures S2 and S3: The 13C SSNMR spectra of films: Table S1: Comparison of strain-to-failure and toughness of the ANF_DCM-W_ film with previously reported. Figure S4: Decomposed C 1s, N 1s, and O1s spectra of films. Tables S2–S5: The chemical composition of films from XPS analysis. Tables S6–S8: Atomistic coordinates for samples. Figure S5: FTIR spectra of films. Figures S6 and S7: SEM images and statistical analysis of diameter distribution of samples. Figure S8: Images of gelation of samples. Figure S9: The UV absorption spectra. Figure S10: TGA and DSC curves of samples. References [6,12,24,25,26,27,28,29,30,31] are cited in the supplementary materials.
