# Supplementary material for: Preparation of Mechanically Strong Aramid Nanofiber Gel Film with Surprising Entanglements and Orientation Structure Through Aprotic Donor Solvent Exchange
Source: Materials (Basel). 2025 Mar 4;18(5):1142. doi: 10.3390/ma18051142 (PMC11901970; doi:10.3390/ma18051142)
Supplement: Supplementary file 1 [file materials-18-01142-s001.zip › materials-3468198-supplementary.pdf]

**Supplementary File**

# Preparation of Mechanically Strong Aramid Nanofiber Gel Film with Surprising Entanglements and Orientation Structure Through Aprotic Donor Solvent Exchange

Zeyu Chen, Chuying Yu, Wenbin Zhong *

College of Materials Science and Engineering, Hunan University, Changsha, 410082, China

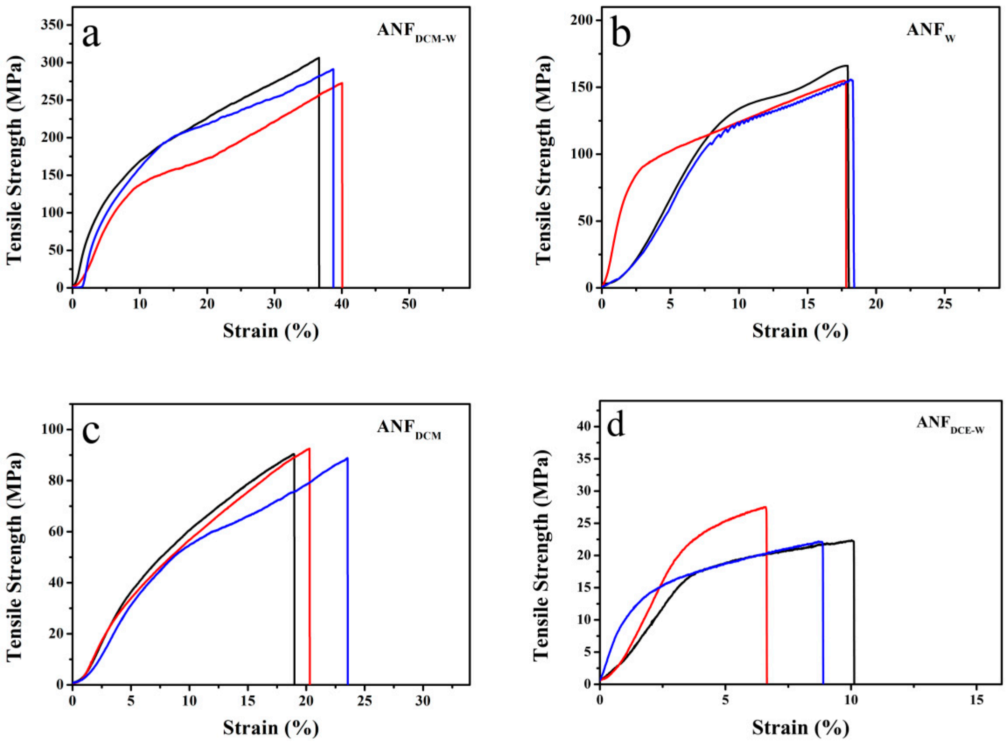


**Figure S1**. Tensile stress-strain curves of (a) ANF$_{DCM-W}$, (b) ANF$_W$, (c) ANF$_{DCM}$ and (d) ANF$_{DCE-W}$

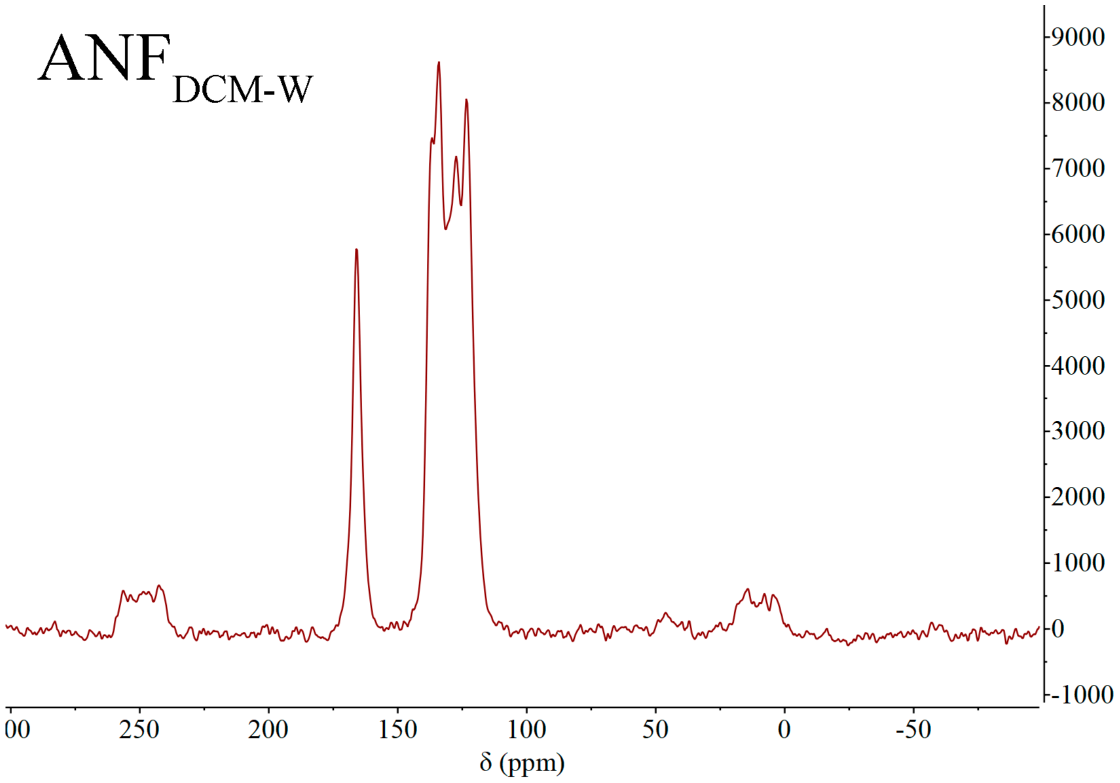


**Figure S2.** The $^{13}C$ SSNMR spectrum of $ANF_{DCM\text{-}W}$.

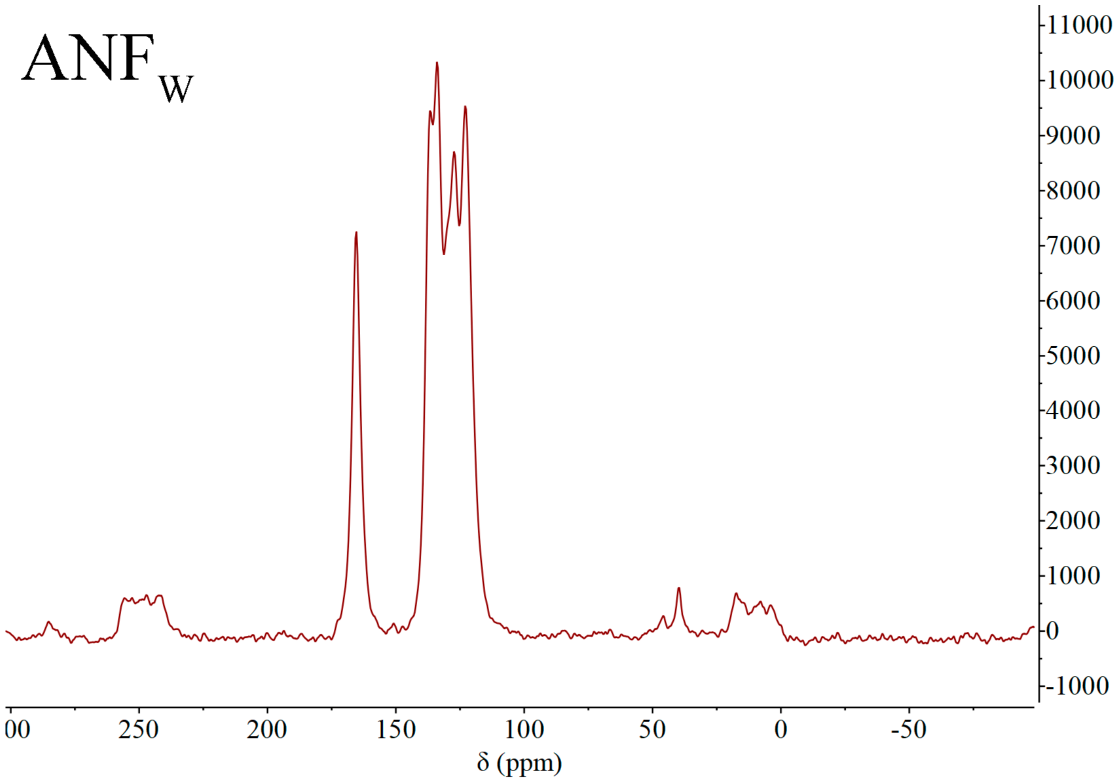


**Figure S3.** The $^{13}C$ SSNMR spectrum of ANFw

**Table S1.** Comparison of strain-to-failure and toughness of the $ANF_{DCM-W}$ film with previously reported.

| Materials | Re-protonated reagent | Tensile Strength σ(MPa) | Toughness (MJ·m$^{-3}$) | Ref. |
|---|---|---|---|---|
| Pure cANFs | water | 146 | ~15 | [S1] |
| Pure ANFs | water | 255.1 | 30.9 | [S2] |
| Pure ANFs | water | 196.1 | ~28 | [S3] |
| Pure ANFs | water | ~160 | ~5 | [S4] |
| Pure ANFs | water | 194.3 | ~12 | [S5] |
| Pure ANF | polyacrylic acid | 267 | / | [S6] |
| Pure ANFs | water | ~200 | ~35 | [S7] |
| Pure ANFs | water | 160 | 37 | [S8] |
| Pure ANFs | water | 187.3 | ~12 | [S9] |
| Aramid Nanofiber/Carbon Nanotube Nanocomposites | Water | 361.2 | / | [S10] |
| mica-based nanopaper with 3D aramid nanofiber framework | Water | 175 | 109 | [S7] |

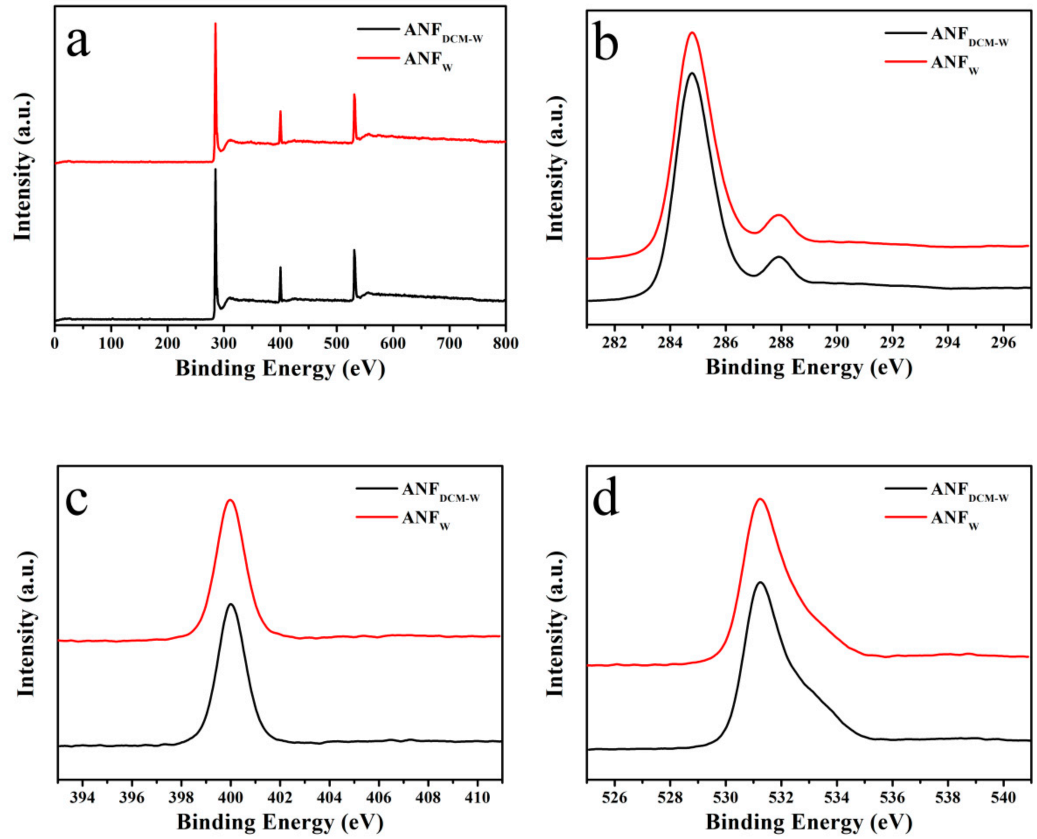


**Figure S4.** (a) The XPS spectra of $ANF_{DCM-W}$ and $ANF_{W}$, (b) C1s spectra of $ANF_{DCM-W}$ and $ANF_{W}$, (c) N1s spectra of $ANF_{DCM-W}$ and $ANF_{W}$, (d) O1s spectra of $ANF_{DCM-W}$ and $ANF_{W}$.

**Table S2.** The chemical composition of $ANF_{DCM-W}$ and $ANF_{W}$ from XPS analysis.

| Sample | C (at.%) | N (at.%) | O (at.%) |
|---|---|---|---|
| $ANF_{DCM-W}$ | 61.46 | 19.88 | 18.6 |
| $ANF_{W}$ | 61.85 | 19.70 | 18.45 |

**Table S3.** Decomposed C 1s energy state of $ANF_{DCM-W}$ and $ANF_{W}$ film from XPS analysis.

| Sample | C-C (284.8 eV) | C-N (285.7 eV) | C=O (287.9 eV) | COOH (290.1 eV) |
|---|---|---|---|---|
| $ANF_{DCM-W}$ | 74.72 | 12.79 | 8.85 | 3.63 |
| $ANF_{W}$ | 72.72 | 15.75 | 7.60 | 3.94 |

**Table S4.** Decomposed N 1s energy state of $ANF_{DCM-W}$ and $ANF_{W}$ film from XPS analysis.

| Sample | C-N (399.9 eV) | N-H (399.2 eV) |
|---|---|---|
| $ANF_{DCM-W}$ | 93.38 | 6.62 |
| $ANF_{W}$ | 95.40 | 4.60 |

**Table S5.** Decomposed O 1s energy state of $ANF_{DCM-W}$ and $ANF_W$ film from XPS analysis

| Sample | C=O of amide (531.2 eV) | C=O of carboxylic acid (531.6 eV) | C-O of carboxylic acid (533.1 eV) |
|---|---|---|---|
| $ANF_{DCM-W}$ | 39.76 | 34.42 | 25.82 |
| $ANF_W$ | 46.16 | 34.06 | 19.79 |

The high resolution C1s, N1s and O1s spectra of $ANF_{DCM-W}$ and $ANF_W$ film and it decomposed peaks are shown in Figure S4, and the content of decomposed energy state are show in Table S3-S5. The $ANF_{DCM-W}$ and $ANF_W$ film exhibit similar chemical composition, except that $ANF_{DCM-W}$ has a slightly higher content of carboxylic acid than that of $ANF_W$. It may be attributed to the deprotonation of PPTA fibers into low molecular weight PPTA polyanions with carboxyl groups, which increase their solubility in water and dissolve in water during the process of solvent exchange.

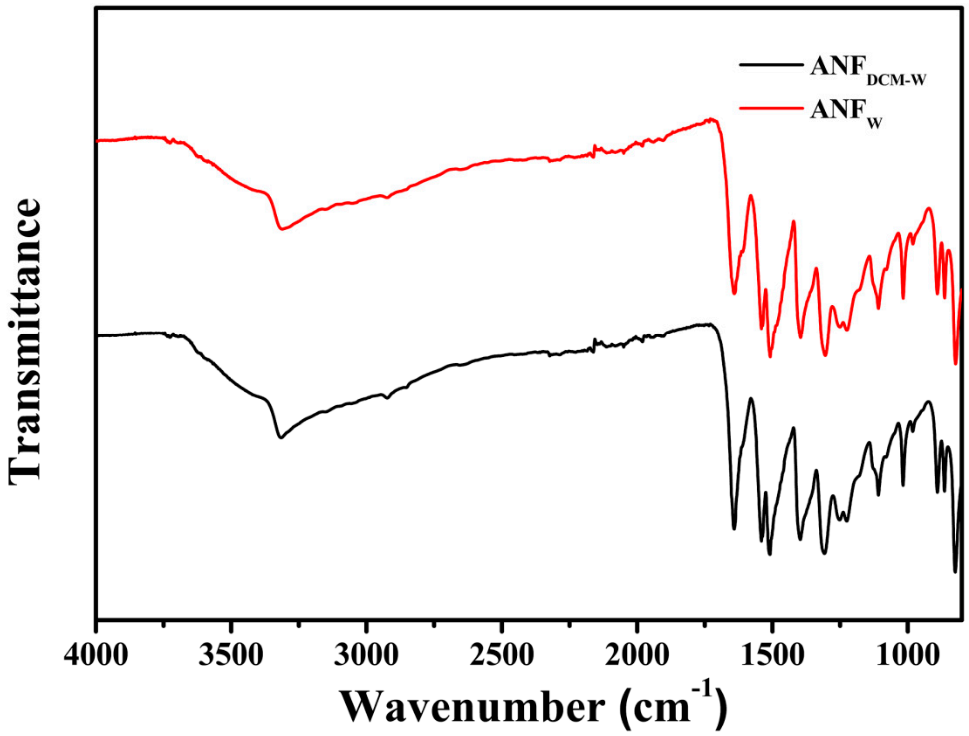


**Figure S5**. The FTIR spectra of ANF$_{DCM-W}$ and ANF$_W$ film.

The peaks at 3317, 1640, 1541, 1510, 1319 and 1016 cm$^{-1}$ are assigned to N-H stretching vibration, C=O stretching vibration of amide group, N-H deformation and C-N stretching coupled modes, C=C stretching vibration of aromatic ring, Ph-N vibration and in-plane C-H vibration, respectively.

**Table S6.** Atomistic coordinates for the PPTA (space group *Pn*, a = 7.87 Å, b = 5.18 Å, c = 12.9 Å, α = β = 90°, γ= 90°)

| Atom | x/a | y/b | z/c |
|---|---:|---:|---:|
| O1 | 0.979 | -0.367 | 0.015 |
| O2 | 1.021 | 0.367 | 0.535 |
| N3 | 1.003 | 0.056 | 0.058 |
| N4 | 0.997 | -0.056 | 0.492 |
| C5 | 1.002 | 0.028 | 0.168 |
| C6 | 1.084 | -0.179 | 0.214 |
| C7 | 1.082 | -0.207 | 0.321 |
| C8 | 0.998 | -0.028 | 0.382 |
| C9 | 0.916 | 0.179 | 0.336 |
| C10 | 0.918 | 0.207 | 0.229 |
| C11 | 1.008 | 0.138 | 0.56 |
| H12 | 1.01354 | 0.25086 | 0.02279 |
| H13 | 0.98646 | -0.25086 | 0.52721 |
| C14 | 0.992 | -0.138 | 0.99 |
| C15 | 0.996 | -0.067 | 0.879 |
| C16 | 1.10817 | 0.12064 | 0.84428 |
| C17 | 1.11222 | 0.18754 | 0.74027 |
| C18 | 1.004 | 0.067 | 0.671 |
| C19 | 0.89183 | -0.12064 | 0.70572 |
| C20 | 0.88778 | -0.18754 | 0.80973 |
| H21 | 1.15329 | -0.32674 | 0.16397 |
| H22 | 1.14957 | -0.37752 | 0.35871 |
| H23 | 0.84671 | 0.32674 | 0.38603 |
| H24 | 0.85039 | 0.37758 | 0.19136 |
| H25 | 1.1973 | 0.2202 | 0.90133 |
| H26 | 1.20462 | 0.34165 | 0.71194 |
| H27 | 0.8027 | -0.2202 | 0.64867 |
| H28 | 0.79538 | -0.34165 | 0.83806 |

**Table S7**. Atomistic coordinates for the $ANF_{DCM-W}$ (space group *Pn*, a = 7.70 Å, b = 4.75 Å, c = 12.9 Å, α = β = 90°, γ= 84°)

| Atom | x/a | y/b | z/c |
|---|---|---|---|
| O1 | 1.04929 | 0.22243 | 0.535 |
| N2 | 1.03079 | -0.11359 | 0.058 |
| N3 | 1.02462 | -0.23469 | 0.492 |
| C4 | 1.02976 | -0.14395 | 0.168 |
| C5 | 1.14476 | -0.34702 | 0.21575 |
| C6 | 1.14334 | -0.37657 | 0.32279 |
| C7 | 1.02565 | -0.20433 | 0.382 |
| C8 | 0.91066 | -0.00127 | 0.33425 |
| C9 | 0.91208 | 0.02829 | 0.22721 |
| C10 | 1.03593 | -0.02504 | 0.56 |
| H11 | 1.04162 | 0.09707 | 0.02279 |
| H12 | 1.01379 | -0.44535 | 0.52721 |
| C13 | 1.01949 | -0.32324 | 0.99 |
| C14 | 1.0236 | -0.24651 | 0.879 |
| C15 | 1.11506 | -0.02536 | 0.847 |
| C16 | 1.11917 | 0.047 | 0.743 |
| C17 | 1.03182 | -0.10177 | 0.671 |
| C18 | 0.94035 | -0.32292 | 0.703 |
| C19 | 0.93624 | -0.39529 | 0.807 |
| O20 | 1.00613 | -0.57071 | 1.015 |
| H21 | 1.24091 | -0.49044 | 0.16717 |
| H22 | 1.23904 | -0.54264 | 0.36199 |
| H23 | 0.81451 | 0.14215 | 0.38283 |
| H24 | 0.81635 | 0.19438 | 0.18804 |
| H25 | 1.18708 | 0.09732 | 0.90629 |
| H26 | 1.19444 | 0.22883 | 0.71693 |
| H27 | 0.86834 | -0.4456 | 0.64371 |
| H28 | 0.86097 | -0.57711 | 0.83307 |

**Table S8**. Atomistic coordinates for the ANFw (space group *Pn*, a = 7.87 Å, b = 5.18 Å, c = 12.9 Å, α = β = 90°, γ= 90°)

| Atom | x/a | y/b | z/c |
|---|---|---|---|
| O1 | 0.479 | 0.133 | -0.015 |
| C2 | 0.492 | 0.362 | 0.01 |
| C3 | 0.496 | 0.433 | 0.121 |
| C4 | 0.6503 | 0.41476 | 0.18117 |
| C5 | 0.64433 | 0.47422 | 0.28587 |
| C6 | 0.504 | 0.567 | 0.329 |
| C7 | 0.3497 | 0.58524 | 0.26883 |
| C8 | 0.35567 | 0.52578 | 0.16413 |
| O9 | 0.521 | 0.867 | 0.465 |
| N10 | 0.503 | 0.556 | 0.942 |
| N11 | 0.497 | 0.444 | 0.508 |
| C12 | 0.502 | 0.528 | 0.832 |
| C13 | 0.42276 | 0.69934 | 0.77164 |
| C14 | 0.42083 | 0.67125 | 0.66464 |
| C15 | 0.498 | 0.472 | 0.618 |
| C16 | 0.57724 | 0.30066 | 0.67836 |
| C17 | 0.57917 | 0.32875 | 0.78536 |
| C18 | 0.508 | 0.638 | 0.44 |
| H19 | 0.51354 | 0.75086 | 0.97721 |
| H20 | 0.48646 | 0.24914 | 0.47279 |
| H21 | 0.77385 | 0.35243 | 0.14241 |
| H22 | 0.76132 | 0.4436 | 0.3365 |
| H23 | 0.22615 | 0.64757 | 0.30759 |
| H24 | 0.23868 | 0.5564 | 0.1135 |
| H25 | 0.3553 | 0.87197 | 0.80784 |
| H26 | 0.35255 | 0.81959 | 0.61439 |
| H27 | 0.6447 | 0.12803 | 0.64216 |
| H28 | 0.6484 | 0.17755 | 0.83372 |

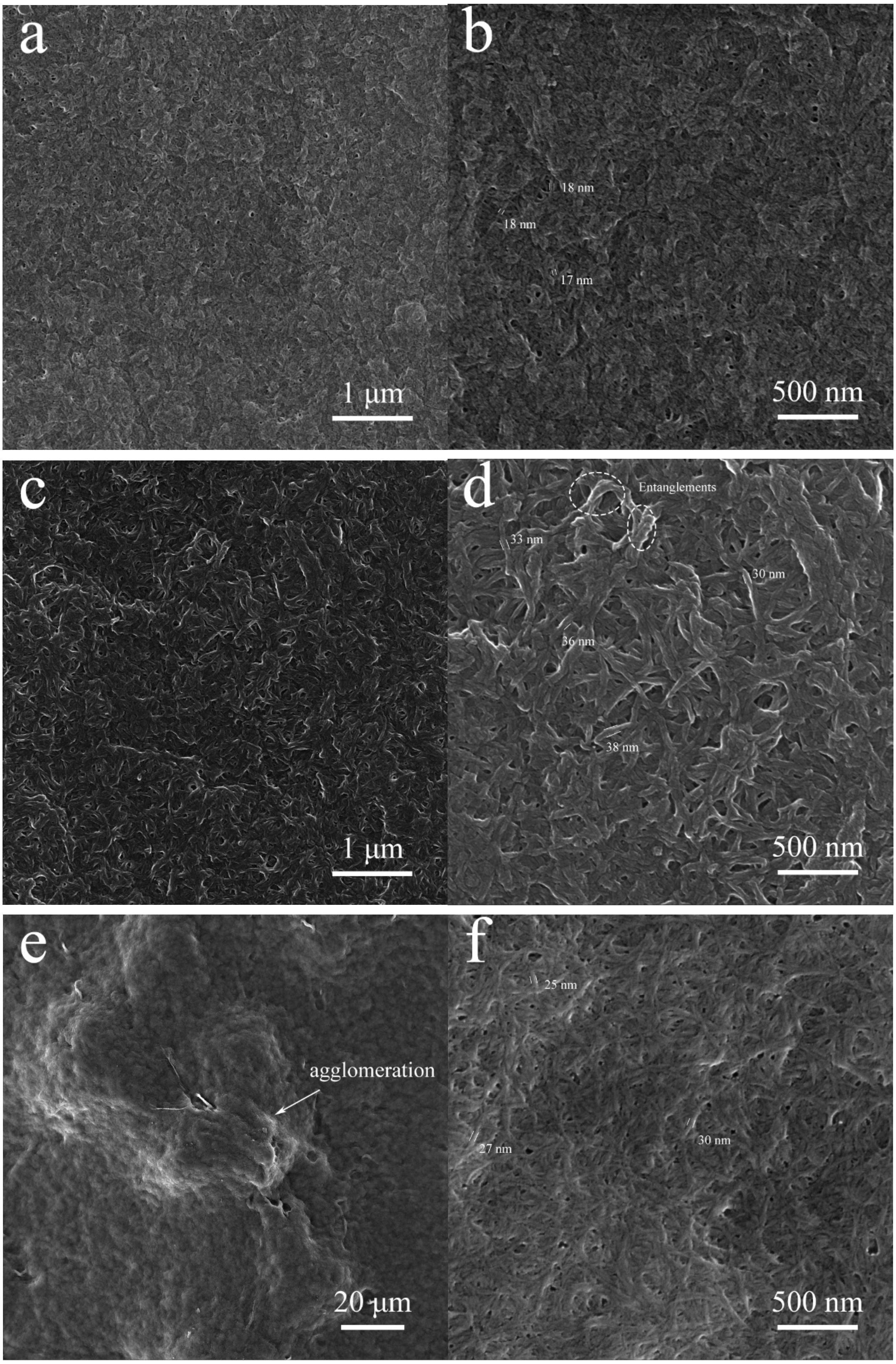


**Figure S6**. SEM images of (a, b) $ANF_W$, (c, d) $ANF_{DCM-W}$, (e, f)$ANF_{DCE-W}$.

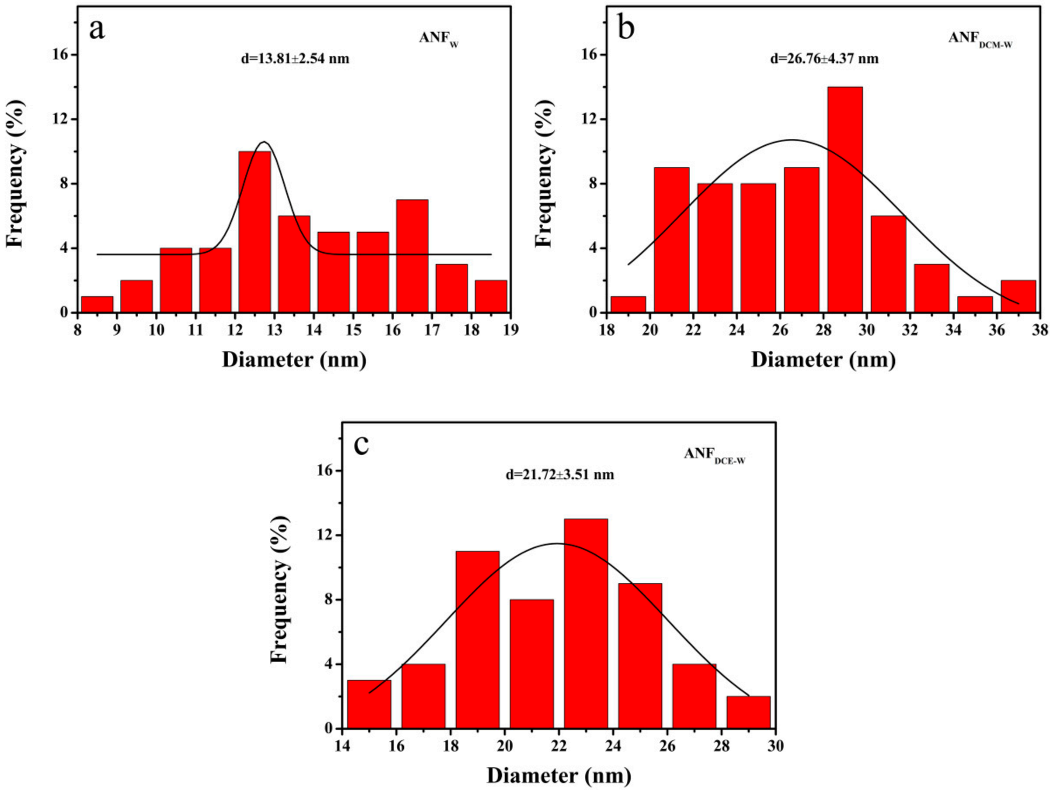


**Figure S7**. Statistical analysis of diameter distribution for films under same conditions as that shown in Figure A4: (a) $ANF_{W}$, (b) $ANF_{DCM-W}$, (c)$ANF_{DCE-W}$.

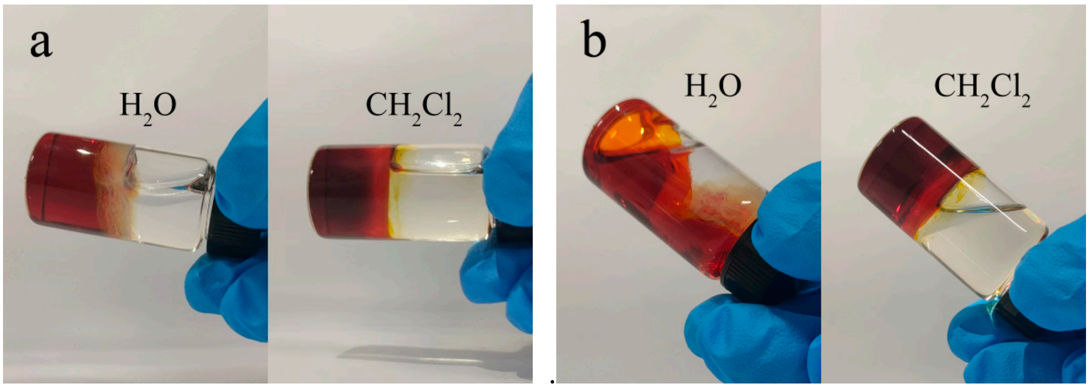


**Figure S8**. The images of ANF$_W$ gel and ANF$_{DCM-W}$ gel tilted by (a) about 90˚, and (b) about 120˚ when the gelation was processed for 2h.

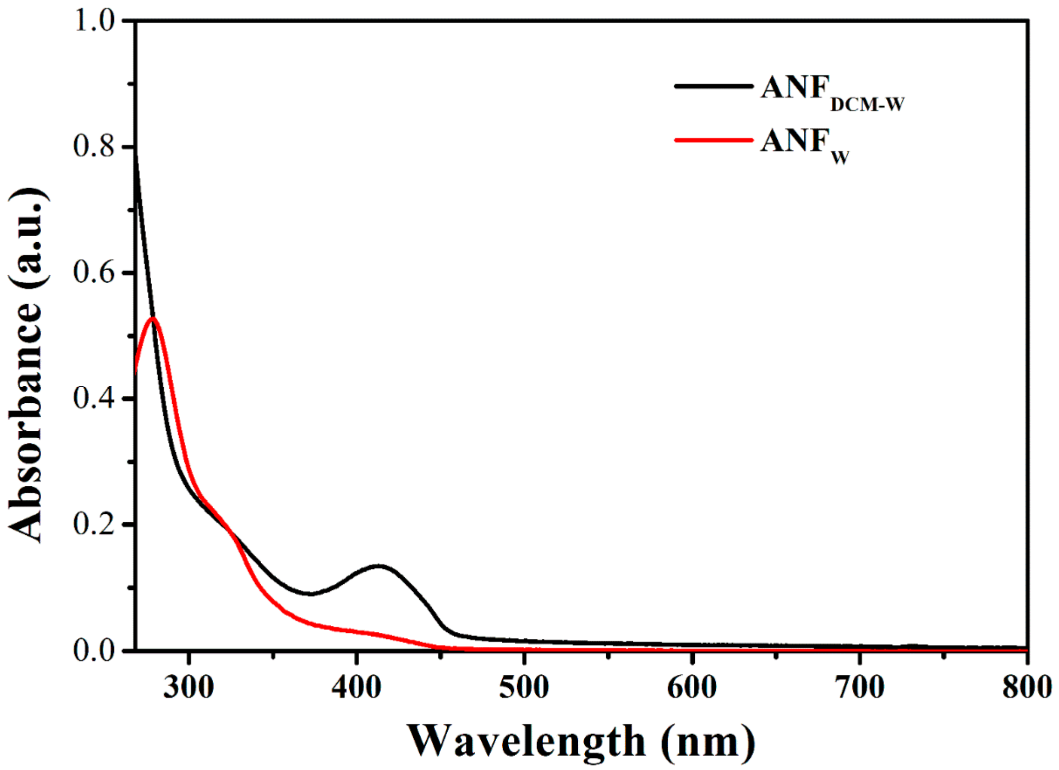


**Figure S9**. The UV absorption spectra of supernatant extracted from ANF$_{DCM}$ and ANF$_{W}$ gel prepared with dichloromethane and water, respectively.

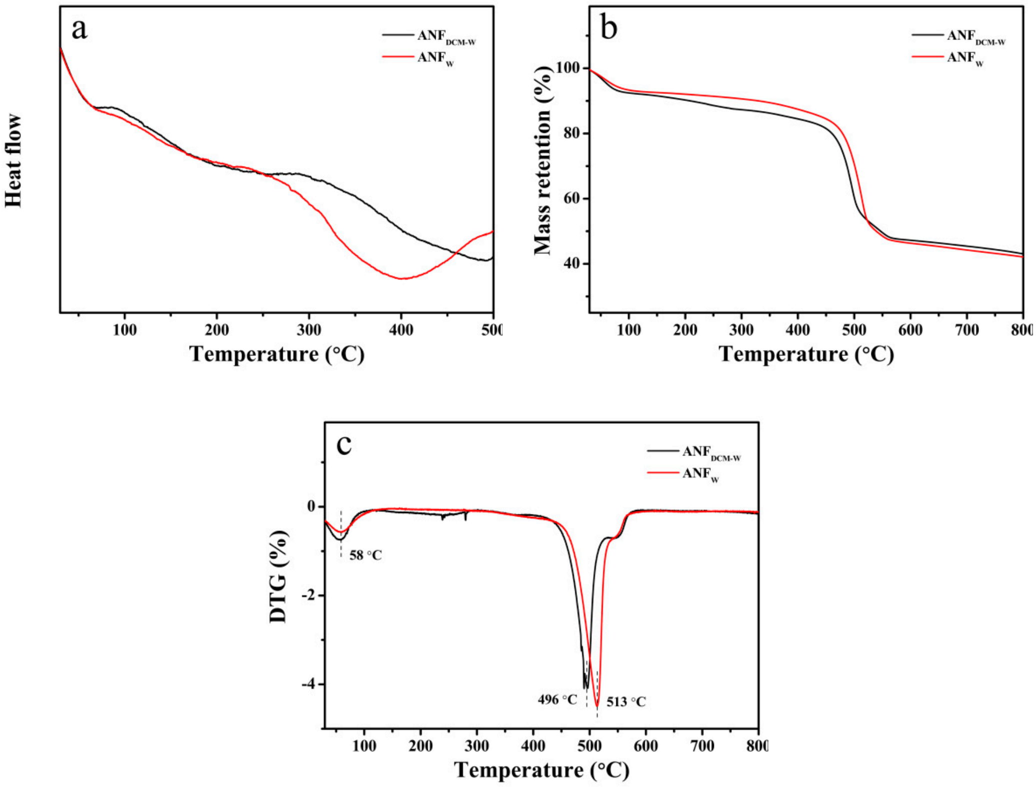


**Figure S10**. The thermal properties of ANF$_{DCM-W}$ and ANF$_W$ film: (a) DSC curves, (b) TGA curves, (c) DTG curves.

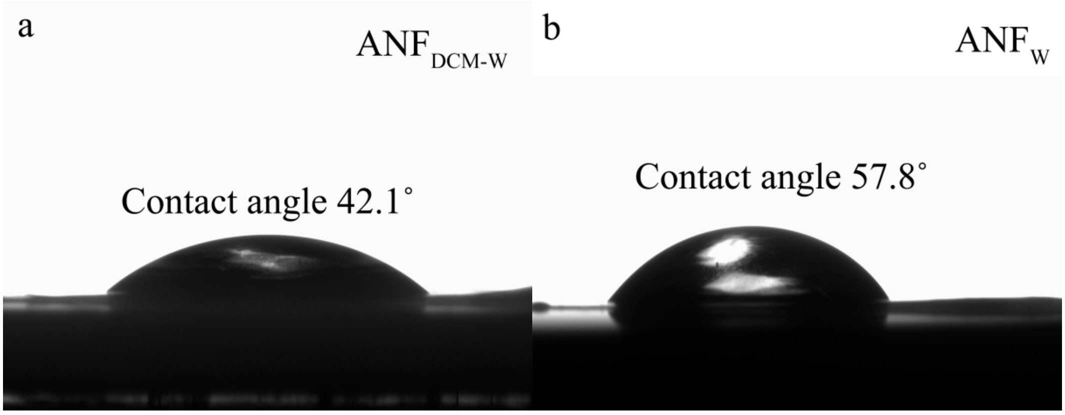


**Figure S11**. The water contact angle of $ANF_{DCM}$ and $ANF_W$ film.

## Reference


[S1] Ma E, S.; Ning Q.; Huang D.; Jin J.; Lu Z. Bio-inspired covalent crosslink of aramid nanofibers film for improved mechanical performances. *Compos. Sci. Technol.* **2021**, *201*, 108514.

[S2] Yin, Q.; Jia, H.; Mohamed, A.; Ji, Q.; Hong, L. Highly flexible and mechanically strong polyaniline nanostructure@aramid nanofiber films for free-standing supercapacitor electrodes. *Nanoscale* **2020**, *12*, 5507–5520.

[S3] Songfeng, E.; Ma, Q.; Huang, J.; Jin, Z.; Lu, Z. Enhancing mechanical strength and toughness of aramid nanofibers by synergetic interactions of covalent and hydrogen bonding. *Compos. Part A Appl. Sci. Manuf.* **2020**, *137*, 106031.

[S4] Wu, K.; Wang, J.; Liu, D.; Lei, C.; Liu, D.; Lei, W.; Fu, Q. Highly thermoconductive, thermostable, and super-flexible film by engineering 1d rigid rod-like aramid nanofiber/2D boron nitride nanosheets. *Adv. Mater.* **2020**, *32*, 1–9.

[S5] Weng, C.; Xing, T.; Jin, H.; Wang, G.; Dai, Z.; Pei, Y.; Liu, L. Mechanically robust anf/mxene composite films with tunable electromagnetic interference shielding performance. *Compos. Part A Appl. Sci. Manuf.* **2020**, *135*, 105927.

[S6] Yang, M.; Cao, K.; Yeom, B.; Thouless, M. D.; Waas, A.; Arruda, E. M.; Kotov, N. A. Aramid nanofiber-reinforced transparent nanocomposites. *J. Compos. Mater.* **2015**, *49*, 1873–1879.

[S7] Zeng, F.; Chen, X.; Xiao, G.; Li, H.; Xia, S.; Wang, J. A bioinspired ultratough multifunctional mica-based nanopaper with 3d aramid nanofiber framework as an electrical insulating material. *ACS Nano* **2020**, *14*, 611–619.

[S8] Xiao, G.; Di, J.; Li, H.; Wang, J. Highly thermally conductive, ductile biomimetic boron nitride/aramid nanofiber composite film. *Compos. Sci. Technol.* **2020**, *189*, 108021.

[S9] Li, H.; Teng, C.; Zhao, J.; Wang, J. A scalable hydrogel processing route to high-strength, foldable clay-based artificial nacre. *Compos. Sci. Technol.* **2021**, *201*, 108543.

[S10] Zhu, J.; Cao, W.; Yue, M.; Hou, Y.; Han, J.; Yang, M. Strong and stiff aramid nanofiber/carbon nanotube nanocomposites. *ACS Nano* **2015**, *9*, 2489–2501.
